# Metabolites Associated with Memory and Gait: A Systematic Review

**DOI:** 10.3390/metabo12040356

**Published:** 2022-04-15

**Authors:** Qu Tian, Brendan A. Mitchell, Abigail E. Corkum, Ruin Moaddel, Luigi Ferrucci

**Affiliations:** 1 Longitudinal Studies Section, Translational Gerontology Branch, National Institute on Aging, Baltimore, MD 21225, USA; brendan.mitchell@nih.gov (B.A.M.); corkumabby@gmail.com (A.E.C.); ferruccilu@grc.nia.nih.gov (L.F.); 2 Laboratory of Clinical Investigation, National Institute on Aging, Baltimore, MD 21224, USA; moaddelru@grc.nia.nih.gov

**Keywords:** metabolomics, memory, gait, dementia

## Abstract

We recently found that dual decline in memory and gait speed was consistently associated with an increased risk of dementia compared to decline in memory or gait only or no decline across six aging cohorts. The mechanisms underlying this relationship are unknown. We hypothesize that individuals who experience dual decline may have specific pathophysiological pathways to dementia which can be indicated by specific metabolomic signatures. Here, we summarize blood-based metabolites that are associated with memory and gait from existing literature and discuss their relevant pathways. A total of 39 eligible studies were included in this systematic review. Metabolites that were associated with memory and gait belonged to five shared classes: sphingolipids, fatty acids, phosphatidylcholines, amino acids, and biogenic amines. The sphingolipid metabolism pathway was found to be enriched in both memory and gait impairments. Existing data may suggest that metabolites from sphingolipids and the sphingolipid metabolism pathway are important for both memory and gait impairments. Future studies using empirical data across multiple cohorts are warranted to identify metabolomic signatures of dual decline in memory and gait and to further understand its relationship with future dementia risk.

## 1. Introduction

Early manifestations of Alzheimer’s disease (AD), the most common form of dementia, include declines in both memory and non-cognitive domains, such as slow gait. Recent data in both aging cohorts and patients with memory complaints seen in the clinic have shown that individuals who experience dual decline in memory and gait have an elevated risk of developing dementia compared to those with memory or gait decline only or no decline [1,2]. However, whether those individuals who experience both mobility and memory decline in the development of AD and related dementias are a subgroup with a specific pathogenetic profile is unknown. We hypothesize that older persons who experience such dual decline undergo metabolic changes that can be revealed as a specific pattern of circulating metabolites.

Metabolomics is an emerging technique that can accurately assess hundreds of metabolites in human biofluids and tissues. The identification of metabolomic signatures of individuals with certain diseases may shed light on underlying mechanisms [3]. The use of readily available biofluids of plasma and serum makes it a simple and cost-effective approach relative to other diagnostic techniques, such as neuroimaging. Two quantitative metabolomics approaches are commonly used, namely nuclear magnetic resonance (NMR) and mass spectroscopy (MS) coupled with liquid or gas chromatography. NMR is highly reproducible with low sensitivity [4]. MS can identify several hundred metabolites with high sensitivity including metabolites with low abundance signals [5]. Metabolomic studies in AD have pinpointed several biological pathways, such as lipid metabolism, methionine, arginine, and glutamate metabolism, fatty acid biosynthesis, mitochondrial bioenergetics [5], sphingolipid transport, saturated fatty acid biosynthesis [6], and cerebral glucose metabolism [7]. Research on metabolites associated with mobility decline may also provide insight into AD pathology since slow gait is considered an early indicator of preclinical AD. Initial studies have suggested that mitochondrial bioenergetics and dysfunction [8,9,10], as well as sphingolipid metabolism (Wennberg et al., 2018), underlie mobility decline.

In this systematic review, we aim to summarize existing data on metabolites associated with memory and gait speed from observational studies and discuss relevant pathways. Findings may provide potential insights into the mechanisms of dual decline in memory and gait speed.

## 2. Materials and Methods

### 2.1. Literature Search and Study Selection

We followed PRISMA guidelines to conduct this systematic review [11]. One author (BAM) searched for literature that was written in English and published after 1 January 1999, using the PubMed database. In the PubMed database, the search terms included (1) “Metabolomics” [Mesh] OR “Metabolite” [tw] OR “Metabolome” [tw], AND (2) “Memory and Learning Tests” [Mesh] OR “Memory” [tw] OR “Cognition” [tw] OR (3) “Walking Speed” [Mesh] OR “Mobility” [tw] OR “Gait” [tw] OR “Physical Function” [tw], AND (4) “Adult” [Mesh]. The PubMed search retrieved 455 records. Twelve additional records that were not shown in the PubMed search were added. These 12 records were discovered in relevant literature or based on author’s knowledge.

**Figure 1:**
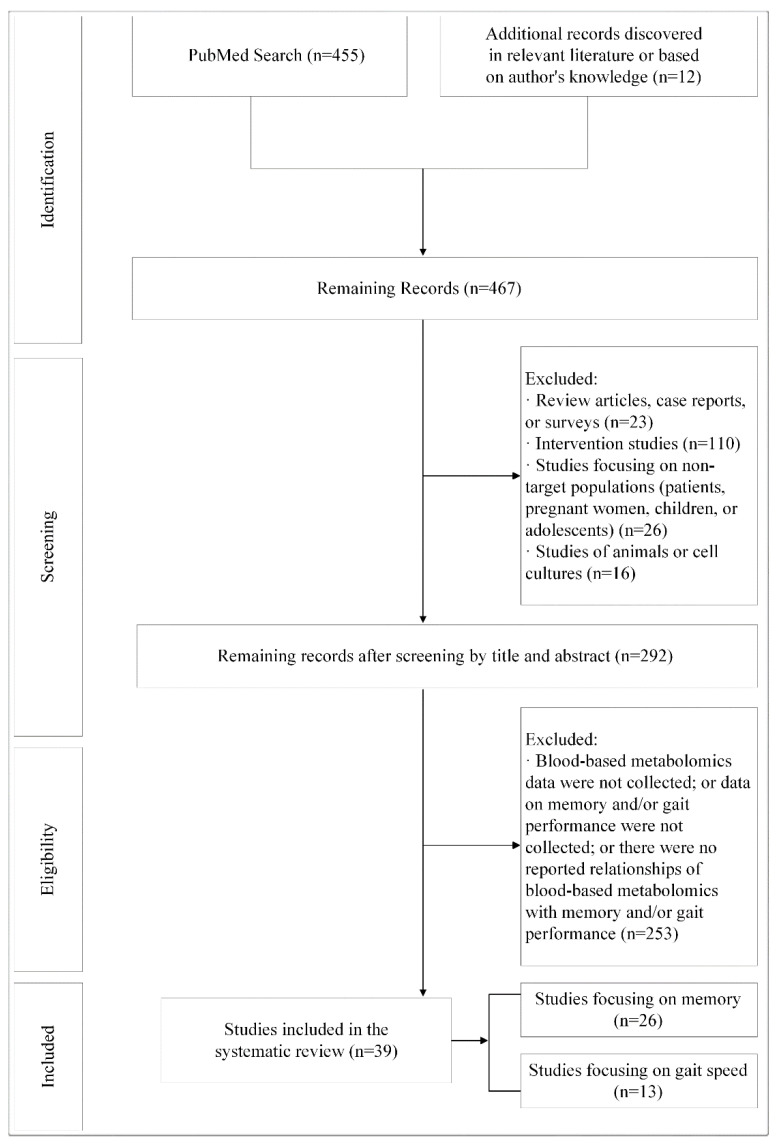
Flow chat of study selection.

Two authors (B.A.M. and Q.T.) evaluated the 467 records. No duplicate records were found. As we focused on observational studies of community-dwelling adults and blood-based metabolomics, we first excluded the following records after screening by title and abstract; reviews, case reports, surveys, intervention studies, and studies of unique populations, animals, and cell cultures. We further excluded studies that met the following exclusion criteria: (1) metabolite data was not blood-based, (2) data on memory and/or gait was not collected, or (3) there were no reported relationships of blood-based metabolomics with memory and/or gait performance. A total of 39 studies were eligible and included in this systematic review (Figure 1).

### 2.2. Analysis

**Figure 2:**
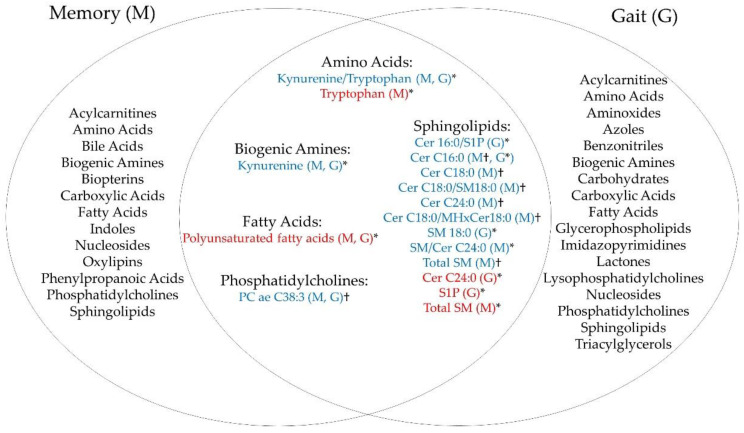
Venn diagram for metabolite classes associated with memory and gait. Legend: Red indicates upregulated metabolites with memory or gait performance. Blue indicates downregulated with memory or gait performance. * = reported cross-sectional associations; † = reported longitudinal associations. Please refer to Table S1 for individual metabolites that were associated with memory only and gait only.

We first summarized key elements of each study, including study cohort, sample size, demographics (age, sex, race/ethnicity, cognitive status), assessment of memory and gait, metabolomics technique, number of metabolites examined, and significance threshold. Details of each study are presented in Table 1 and Table 2, sorted by the number of metabolites examined. We then categorized metabolites that were associated with memory and gait, identified shared metabolite classes (Figure 2 for Venn diagram), and also identified the direction of these associations (Table S1). As the significance threshold varied across studies, we reported results based on the threshold defined in each study.

We further conducted the Kyoto Encyclopedia of Genes and Genomes (KEGG) pathway analysis via https://www.metaboanalyst.ca/ accessed on 3 March 2022. First, we identified metabolite IDs from the Human Metabolome Database (HMDB https://hmdb.ca/ accessed on 3 March 2022) that were associated with memory and gait. Only metabolites with unique HMDB IDs were included for pathway analysis. We then entered HMDB IDs into the KEGG database. Specific parameters were as follows: scatter plot, hypergeometric test, relative-betweenness centrality, and use all compounds in KEGG (homo sapiens) library. We reported significant pathways at *p*-value ≤ 0.05.

## 3. Results

### 3.1. Overview

Out of 39 studies included in this systematic review, 26 reported associations of metabolites with memory and 13 with gait. None of these studies examined associations of metabolites with memory and gait simultaneously. The mean age of these 39 studies was between 26 and 85 years old. Most studies included both men and women (*n* = 34). Two studies examined women only, and 3 examined men only. Study samples were geographically diverse with 18 from North America, 7 from Europe, 7 from Asia, 1 from Oceania, and 6 not specified (Table 1 and Table 2). Most studies were community-based (*n* = 27), 9 studies recruited participants from clinics/hospitals, and 3 had mixed populations. All studies used either quantitative and/or semi-quantitative methods for metabolomics. Metabolomics techniques included various analytical platforms, mostly mass spectroscopy (*n* = 30). Most studies analyzed individual metabolites and 3 included pathway analysis. Out of 39 studies, 13 examined over 100 metabolites. Among 13 studies examining over 100 metabolites, 6 used adjusted *p*-values (mostly FDR-adjusted *p* < 0.05, *n* = 4), 4 used unadjusted *p*-value thresholds, and 3 used both adjusted and unadjusted *p*-value thresholds. Among 26 studies examining less than 100 metabolites, 21 used unadjusted *p*-value thresholds, 4 used adjusted *p*-value thresholds, and 1 used a variable importance measure derived from a random forest algorithm.

We found that metabolites in five shared classes were associated with memory and gait impairments, namely sphingolipids (SLs) (including sphingomyelins (SMs) and ceramides), fatty acids (FAs), phosphatidylcholines (PCs), amino acids (AAs), and biogenic amines (see Figure 2 for Venn diagram).

### 3.2. Metabolites and Memory Performance

Details of the 26 studies reporting associations of metabolites with memory are summarized in Table 1. The assessment of memory function varied, including verbal memory, working memory, visual memory, episodic memory, and semantic memory. The types of examined biofluids included plasma (*n* = 15) and serum (*n* = 11). Twenty-one studies reported associations in cognitively normal older adults, one in cognitive impairment, and one in AD. Six studies reported associations in persons with mixed cognitive status, including cognitively normal and cognitively impaired or demented. Out of 26 studies, 11 reported longitudinal associations with changes in memory over time.

A list of metabolites that were associated with memory are presented in Table S1. Downregulated metabolites (i.e., lower concentrations associated with higher memory performance) were found in classes of AAs, biogenic amines, biopterins, carboxylic acids (CAs), FAs, lipoproteins, PCs, SLs (including SMs and ceramides), and xanthines. Upregulated metabolites (i.e., higher concentrations associated with higher memory performance) were found in classes of AAs, acylcarnitines, CAs, FAs, lipoproteins, PCs, SLs (SMs), and xanthines.

### 3.3. Metabolites and Gait

Details of the 13 studies focusing on gait are summarized in Table 2. Gait speed was measured over 6 m (*n* = 4), 5.6 m (*n* = 1), 4 m (*n* = 2), 15 ft (*n* = 2), 8 ft (*n* = 1), 20 m (*n* = 1), over 6 min (*n* = 1), and by the Timed Up and Go Test (3 m away) (*n* = 1). The types of examined biofluids included plasma (*n* = 10) and serum (*n* = 3). Ten studies reported associations in cognitively normal older adults, and 3 studies reported associations in persons with mixed cognitive status, including cognitively normal and cognitively impaired or demented. Out of 13 studies, 2 reported longitudinal associations with changes in gait or mobility over time.

A list of metabolites that were associated with gait are presented in Table S1. Downregulated metabolites (i.e., lower concentrations associated with faster gait speed) were found in classes of FAs, acylcarnitines, carbohydrates, AAs, SLs (SMs and ceramides), CAs, lysoPCs (LPCs), nucleosides, biogenic amines, and azoles. Upregulated metabolites (i.e., higher concentrations associated with faster gait speed) were found in classes of FAs, LPCs, AAs, PCs, CAs, nucleosides, benzonitriles, carbohydrates, glycerophospholipids, triacylglycerols, and SLs (SMs and ceramides).

### 3.4. Pathway Analysis

**Figure 3:**
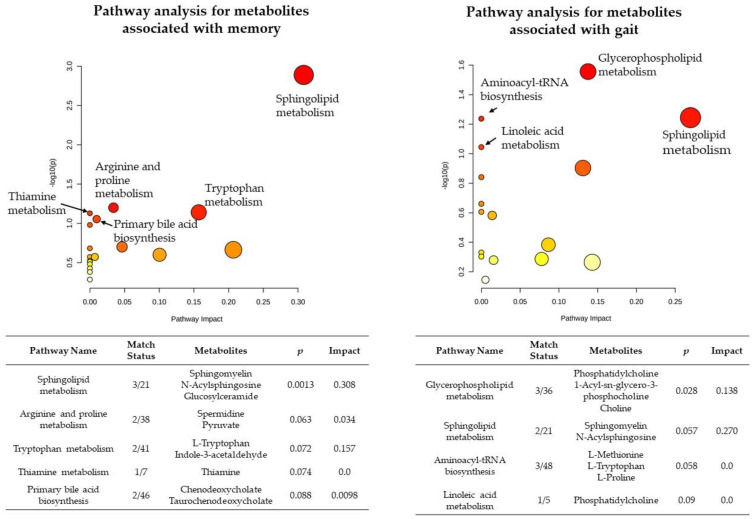
Pathway analysis for metabolites associated with memory and gait.

Memory-related metabolites were enriched in the sphingolipid metabolism pathway (*p* < 0.05) (Figure 3). Other pathways with a trend toward significance included arginine and proline metabolism, tryptophan metabolism, thiamine metabolism, and primary bile acid biosynthesis (0.05 < *p* < 0.10) (Figure 3).

Gait-related metabolites were enriched in pathways of glycerophospholipid metabolism, aminoacyl-tRNA biosynthesis, and sphingolipid metabolism (*p* ≤ 0.05) (Figure 3). The linoleic acid metabolism pathway showed a trend (0.05 < *p* < 0.10) (Figure 3).

## 4. Discussion

In this review, we found five shared metabolite classes associated with both memory performance and gait speed. The majority of metabolites associated with memory and gait were sphingolipids and fatty acids. Metabolites from several classes, including sphingolipids, fatty acids, and amino acids, showed similar directions of the associations with memory and gait (both upregulated or downregulated). Metabolites from classes of amino acids and biogenic amines showed some differences in directions of the associations with memory and gait. The novelty of this work is to systematically identify shared metabolites and pathways related to both memory and gait impairments. A comprehensive review on related metabolites may provide insight into mechanisms underlying dual decline in relation to high dementia risk. To address the specific topic of the dual decline in memory and gait, we focused on reported metabolite classes that were associated with both memory and gait impairments. Here we discuss the shared metabolite classes and relevant pathways and focus on two main classes–sphingolipids and fatty acids.

Metabolites from sphingolipids and the sphingolipid metabolism pathway were found to be shared between memory and gait, many of which were long-chain ceramides. This is in line with previous humans and animal studies which suggest that alterations to the ceramide/sphingosine-1-phosphate rheostat ratio may contribute to the aging process, where ceramides contribute to cellular senescence and sphingosine-1-phosphate delays it [51,52]. Additional evidence suggests a balance between long (e.g., C16:0, C18:0, C20:0) and very-long-chain (e.g., C24:0 and C24:1) ceramides is important for regulating intrinsic cell apoptosis and proliferation [53,54]. Further, ceramide accumulation has been implicated in pro-inflammatory actions and can increase skeletal muscle insulin resistance [55,56]. Notably, elevated levels of ceramide promote β-amyloid production [57], and β-amyloid can, in turn, promote ceramide formation [58]. β-amyloid is not only a hallmark of Alzheimer’s disease but is also associated with impaired mobility in older adults [59,60,61]. We hypothesize that impaired sphingolipid metabolism may be a potential mechanism for memory and gait decline.

The biosynthesis of the unsaturated fatty acids pathway may also play a key role in both memory and gait decline. We found that various types of unsaturated fatty acids, such as poly-unsaturated fatty acids and omega-3 fatty acids, were associated with memory and gait. Previous studies have suggested that omega-3 fatty acids, namely DHA and EPA, may have independent and complementary neuroprotective effects in aging and AD, such as controlling apoptotic mechanisms, combatting amyloid-β production and plaque deposition, and anti-inflammatory derivatives [62,63,64]. Arachidonic acid and its derivatives are largely pro-inflammatory, opposing the effects on inflammatory signaling by omega-3 fatty acids [65,66]. This is in line with several rodent studies showing dietary-induced deficiencies in omega-3 fatty acids and elevations in the AA/(EPA + DHA) ratio leading to increased pro-inflammatory cytokines [67,68].

Besides sphingolipids and fatty acids, amino acids and the kynurenine pathway may also play key roles in both memory and gait decline. For instance, amino acids are the building blocks for protein synthesis. Since amino acids are involved in multiple physiological processes in the body, such as the cell building and synthesis of neurotransmitters, they play key roles in skeletal muscle function and brain function [69,70]. The kynurenine-tryptophan metabolism pathway contributes to mitochondrial dysfunction and inflammation which may affect aging phenotypes. Kynurenine and related metabolites are associated with impaired mitochondrial function and oxidative stress, which can lead to cellular damage and increase inflammation [71,72,73].

In conclusion, existing data suggests that five metabolite classes (amino acids, biogenic amines, fatty acids, PCs, and SMs) are implicated in both memory and gait impairments, with sphingolipids having the largest number of metabolites. Notably, some metabolite classes, such as triacylglycerols, were only studied in gait impairment. Since these classes have not been studied in both impairments, whether they are informative in delineating the metabolomic signature of dual decline remains unclear. Future studies should consider establishing a rigorous scientific protocol across multiple aging cohorts to understand which metabolites and pathways underlie dual decline in memory and gait.

## Figures and Tables

**Table 1:** Summary of studies examining relationships of metabolites with memory (*n* = 26).

Study Name (First Author, Year)	*n* (Women%) Age, Mean (SD), Median (IRQ), Range, Cognitive Status	Race/Ethnicity (%)	Memory Assessment	Metabolomics Technique	Sample Type; Number of Metabolites Analyzed (Classes)	Threshold for Statistical Significance
Bogalusa Heart Study (Shi et al., 2019) * [12]	*n* = 1177, 59.7% 48.11 (5.26)	White (65%) and Black (35%)	WAIS-IV for working memory and WMS-IV for verbal memory	UPLC-MS/MS (Metabolon Inc., Durham, NC, USA)	Serum 1466 (1202 analyzed, including AAs, FAs, carbohydrates, and nucleotides)	FDR (Bonferroni correction); *p* < 4.16 × 10^−5^ (=0.05/1202)
WRAP (Darst et al., 2021) † [13]	*n* = 2324, 68.8% 62 (6.8), range 40–81	White and non-Hispanic (95%)	Composite score for delayed recall from RAVLT, WMS-R LM, and BVMT-R	UPLC-MS/MS (Metabolon Inc., Durham, NC, USA)	Plasma 1097 (untargeted, including AAs, FAs, carbohydrates, and nucleotides)	FDR (Benjamini-Hochberg correction); *q* < 0.05
EMIF-AD Multimodal Biomarker Discovery Study (Kim et al., 2019) * [14]	*n* = 593, 53% CN: 65.06 (7.93) MCI: 70.44 (7.86) AD: 69.55 (8.51)	Not specified (European, 100%)	AVLT for immediate and delayed verbal memory	UPLC-MS/MS (Metabolon Inc., Durham, NC, USA)	plasma 883 (648 analyzed, then focused on only 9, including AAs and FAs)	FDR (Bonferroni correction); *p* < 7.72 × 10^−5^ (=0.05/648)
Mental Health Center of West China Hospital, Sichuan University (Du et al., 2021) * [15]	*n* = 83 (controls); 62.7% 26.4 (8.62), range 18–60	East Asian (Chinese, 100%)	Neuropsychological Tests Automated Battery for spatial working memory	LC-MS/MS	plasma 728 (296 analyzed, including AAs, acylcarnitines, biogenic amines, carbohydrates, LPCs, and PCs)	Spearman rank correlation *p* < 0.05
Outpatient Dialysis Clinics in Northern California (Kurella Tamura et al., 2016) * [16]	*n* = 141, 36% 56.6 (14.6)	White (42.6%)	Controlled Oral Word Association for verbal memory and language and RAVLT for delayed recall.	GC & LC-MS/MS (Metabolon Inc., Durham, NC, USA)	plasma 562 (95 analyzed, including AA derivatives)	FDR (Benjamini-Hochberg correction) *q* < 0.05
MRC NSHD British 1946 Birth Cohort (Proitsi et al., 2018) *,† [17]	*n* = 909; 52% range 60–64	Not specified (British: English, Scottish, and Welsh, 100%)	Three-trial 15-item word list learning task for short-term verbal memory and an uncued delayed free recall trial.	NMR	serum 233 (including FAs, and AAs)	Multiple testing correction; *p* < 0.002 (=0.05/principal components)
Community-Dwelling African American Participants in the Biracial ARIC study (Bressler et al., 2017) † [18]	*n* = 1534 (*n* = 1393 without incident dementia); 63.6% 53.4 (5.8), range 45–64	Black (African American, 100%)	DWRT for verbal memory	GC/MS and LC-MS (Metabolon Inc., Durham, NC, USA)	serum 204 (including AAs and FAs)	FDR (Dubey/Armitage-Parmar correction); *p* < 3.9 × 10^−4^
Rochester/Orange County Aging Study (Mapstone et al., 2017) * [19]	*n* = 224, 62% superior memory: 83.2 (3.4) normal control: 82.3 (3.6) MCI/AD: 81.9 (4.4)	Not specified	RAVLT for verbal memory	Triple quadrupole MS, SID-MRM-MS, and FIA MS/MS (Biocrates, Innsbruck, Austria, p180)	plasma 188 (185 analyzed, then focused on only 12, including AAs, acylcarnitines, PCs, LPCs, SLs, and biogenic amines)	*p* < 0.05
ARIC study (Li et al., 2016) * [20]	*n* = 441, 54.42% CN: 77.6 (5.5) MCI: 76.5 (5.6) Dementia: 79.7 (5.1)	Black (African American, 85.1%)	Delayed word recall, logical memory test part A and B, and incidental learning	triple-quadrupole MS (Biocrates, Innsbruck, Austria, p180)	Plasma 188 (main analysis focused on 9 metabolites including PCs and LPCs; additional analyses explored 151)	*p* < 0.05 for 9 metabolites in main analysis. FDR (Bonferroni correction) for 151 metabolites in exploratory analysis *p* < 0.00033 (=0.05/151)
BLSA (Varma et al., 2018) *,† [21]	*n* = 207, 51.69% 78.68 (7.23)	White (83.09%)	CVLT for learning and immediate and long delay free recall	FIA-MS/MS and HPLC-MS/MS (Biocrates, Innsbruck, Austria, p180)	serum 187 (20 analyzed including AAs, SLs, PCs, acylcarnitines, and biogenic amines)	*p* < 0.05
ROS and MAP (Huo et al., 2020) † [22]	*n* = 530, 78.5% 82 (7.4)	White (European origin, 100%)	episode, working, and semantic memory	FIA-MS/MS and UHPLC-MS/MS (Biocrates, Innsbruck, Austria, p180)	serum 182 (including AAs, biogenic amines, acylcarnitines, PCs, and SLs)	FDR (Benjamini-Hochberg correction); *q* ≤ 10%
Sunnybrook Hospital (Sylvestre et al., 2020) * [23]	*n* = 18 (controls); 66.7% 48.7 (7.2)	Not specified (Canadian, 100%)	BVMT-R for visuospatial memory	1H-NMR spectroscopy	plasma 56 (9 analyzed, mostly AAs)	Spearman’s rank correlation, *p* < 0.05; post-hoc FDR (Bonferroni correction)
Stroke Prevention Clinic (Yu et al., 2019) * [24]	*n* = 25 (healthy controls with minimal SIVD); 54% 71.7 (7.9), range 50–85	Not specified (Canadian, 100%)	CVLT-II for verbal memory (short delayed free recall, long-delayed recall, and recall discriminability)	UPLC-MS/MS	serum Not specified (24 analyzed, oxylipins only)	FDR (Bonferroni correction)
Hordaland Health Study (Solvang et al., 2019) * [25]	*n* = 2174, 55.2% median 71, range 70–72	Not specified (Norwegian, 100%)	KOLT for immediate recall and COWAT for verbal memory	LC-MS/MS	plasma 12 (targeted, AAs and biogenic amines only)	FDR (Bonferroni correction); *p* < 0.0042 (=0.05/12)
WHAS II (Mielke, Bandaru et al., 2010) *,† [26]	*n* = 100 (100%) 74 (2.5), 70–79	Black (African American, 23%)	HVLT-R for verbal immediate and delayed recall	ESI/MS/MS	serum Not specified (12 analyzed, including SLs and cholesterols)	*p* < 0.05
Josep Trueta University Hospital (Arnoriaga et al., 2020) * [27]	*n* = 116; 69.8% median 50.4, IQR: 41.8–58.5	Not specified (Spanish, 100%)	CVLT for immediate and short delayed recall and TDS for working memory	LC-MS/MS (Scharlau, Barcelona, Spain)	plasma Not specified (untargeted, including AAs, FAs, Indoles, and Phenylpropanoic acids)	Variable importance measure from random forest algorithm
Living Cohort (Kindler et al., 2020) * [28]	*n* = 81 (healthy controls); 50.6% 31.7 (8.5)	Not specified (Australian, 100%)	WAIS-III LNS for working memory and WMS-R LM for verbal memory	UHPLC and GC-MS (Agilent, Santa Clara, CA, USA)	plasma Not specified (targeted, kynurenine pathway metabolites only)	*p* < 0.05
ROS and MAP (Borkowski et al., 2021) * [29]	*n* = 198 (59 fasted); 88% 78.2 (7.2)	White and non-Hispanic (95%)	Global measures of episodic, semantic, and working memory from 17 tests	LC-MS/MS	serum Not specified (targeted, lipid mediators only)	Spearman’s rank correlation, *p* < 0.05
Community-Dwelling Volunteers Recruited From the Clinical Core of the Johns Hopkins Alzheimer’s Disease Research Center (Mielke, Haughey et al., 2010) * [30]	*n* = 63; 39.7%; CN: 74.4 (7.0) MCI: 74.5 (5.6) AD: 74.8 (7.0) All 55+	White (96%)	CVLT for verbal memory and Logical Memory Story A from the Wechsler Memory Scale for immediate and delayed recall.	HPLC/MS/MS	plasma 8 SLs (2 analyzed, Cer only)	*p* < 0.05
Cardiac Rehab Program at the Rumsey Centre of University Health Network Toronto Rehab Institute (Chan et al., 2018) † [31]	*n* = 60, 16.7% 64.6 (6), range 50–75 46 CN and 14 with possible MVND (sMMSE <24 excluded); all had CAD	White (79.7%)	CVLT-II for verbal memory and BVMT-R for visuospatial memory.	LC/MS/MS	plasma Not specified (5 analyzed, including SLs)	*p* ≤ 0.05
Sensory-cognitive and Physical Fitness Training in Mild Cognitive Impairment Study (Küster et al., 2017) *,† [32]	*n* = 47, 57.4% 71.2 (6), range 60–88	Not specified (German, 100%)	German CVLT for verbal memory and Everyday Cognition Battery for working memory	Enzyme-linked Immunosorbent Assay kit (Promega Corporation, Madison, WI, USA), spectrophotometer, and LC-MS/MS	serum 6 (targeted, mostly kynurenine pathway metabolites)	*p* < 0.05
BLSA (Simpson et al., 2016) *,† [33]	*n* = 107, 39.25% 72.92 (7.61)	Not specified	CVLT for verbal memory in short and delayed recall tests. BVRT for visual memory.	UPLC-MS	plasma 3 (targeted, PCs only)	*p* < 0.005
WHAS II (Mielke et al., 2008) † [34]	*n* = 426, 100% 74.5 (2.8), range 70–79	Black (African American, 19%)	HVLT-R for verbal immediate and delayed memory	Total/HDL cholesterol levels were calculated using standard enzymatic techniques. LDL calculated using Friedewald equation.	serum Not specified (3 analyzed, including FAs and cholesterols)	*p* < 0.05
Karolinska Schizophrenia Project (Becklén et al., 2021) * [35]	*n* = 22 (healthy controls); 50% median 25, IQR: 22–28	Not specified (Swedish, 100%)	WMSIII for working memory: Spatial Span and Letter-Number Span	Colorimetry (Roche Diagnostics, Basel, Switzerland)	plasma 1 (targeted, bilirubin only)	Spearman’s rank correlation, *p* < 0.05
Kaohsiung Chang Gung Memorial Hospital (Wang et al., 2018) * [36]	*n* = 65 (healthy controls); 44.6% 40.1 (12), range 18–65	East Asian (Chinese, 100%)	List Learning Test for verbal memory and Digit Sequencing Task for working memory	MicroMolar Cysteine Assay Kit (ProFoldin, Hudson, MA, USA)	serum 1 (targeted, cysteine only)	*p* < 0.05
HANDLS Study (Beydoun et al., 2016) *,† [37]	*n* = 2630, 56.6% 47 (0.3), range 30–64	Not specified	CVLT for immediate and delayed free recall and BVRT for visual memory.	Spectrophotometry (Quest Diagnostics, Secaucus, NJ, USA)	serum Not specified (1 analyzed, uric acid only)	FDR (Bonferroni correction for multiple cognitive tests); *p* < 0.004 (=0.05/11)

Notes: SD = standard deviation. IRQ = interquartile range. AA = amino acid. FA = fatty acid. FDR = false discovery rate. WHAS = Women’s Health and Aging Study; BLSA = Baltimore Longitudinal Study of Aging; MRC = Medical Research Council; NSHD = National Survey of Health and Development; ROS = Religious Orders Study; MAP = Rush Memory and Aging Project; ARIC = Atherosclerosis Risk in Communities; WRAP = Wisconsin Registry for Alzheimer’s Prevention; HVLT-R = Hopkins Verbal Learning Test Revised; CVLT = California Verbal Learning Test; BVMT-R = Brief Visuospatial Memory Test Revised; DWRT = Delayed Word Recall Test; BVRT = Benton Visual Retention Test; RAVLT = Rey Auditory Verbal Learning Test; AVLT = Auditory Verbal Learning Test; KOLT = Kendrick Object Learning Test; COWAT = Controlled Oral Word Association Test; WMS = Wechsler Memory Scale; WMS-R LM = WMS-Revised Logical Memory; WAIS = Weschler Adult Intelligence Scale; LNS = Letter-Number Sequencing; SIVD = Subcortical Ischemic Vascular Disease; CAD = coronary artery disease. MCI = Mild Cognitive Impairment; AD = Alzheimer’s Disease; ESI = Electrospray Ionization; MS = Mass Spectrometry; LC = Liquid Chromatography; HPLC = High Performance LC; HDL = High-Density Lipoprotein; LDL = Low-Density Lipoprotein; GC = Gas Chromatography; FIA = Flow Injection Analysis; UPLC = Ultra Performance LC; NMR = Nuclear Magnetic Resonance; UHPC = Ultra-High-Performance Concrete; SID-MRM = Stable Isotope Dilution Multiple Reaction Monitoring; HANDLS = Healthy Aging in Neighborhoods of Diversity across the Life Span; EMIF-AD = European Medical Information Framework for Alzheimer’s Disease; sMMSE = Standardized Mini-Mental State Examination; TDS = Total Digit Span; * = reported cross-sectional associations; † = reported longitudinal associations. Studies are sorted by the number of metabolites examined, from highest to lowest. For the following papers including patient populations, we only reported results and demographics for controls (Sylvestre et al., 2020; Yu et al., 2021; Du et al., 2021; Kindler et al., 2020; Becklén et al., 2021; Wang et al., 2018).

**Table 2:** Summary of studies examining the relationship of metabolites with gait (*n* = 13).

Study Name (First Author, Year)	*n* (Women%) age, Mean (SD), Median (IRQ), Range, Cognitive Status	Race/Ethnicity (%)	Gait Assessment	Metabolomics Technique	Sample Type Number of Metabolites Analyzed (Classes)	Threshold of Significance
Bogalusa Heart Study (Nierenberg et al., 2020) *,† [38]	*n* = 1239; 58.92% 48.2 (5.3)	White (65.5%)	6-minute walk	UPLC-MS/MS (Metabolon Inc. Durham, NC, USA)	serum 1466 (1202 analyzed, including AAs, carbohydrates, FAs, LPCs and SLs)	*p* < 0.05
CHS All Stars Study (Marron et al., 2020) * [39]	*n* = 120, 60% 85(2.9), range 79–95	White (90%)	15 ft walk	LC-MS	plasma 605 (569 analyzed, including AAs and FAs)	*p* < 0.05 and FDR (Benjamini-Hochberg correction); *q* ≤ 30%
Health ABC Study (Murphy et al., 2019) * [40]	*n* = 313, 0% 74.6(2.8), range 70–79	Black (African American, 100%)	20 m usual walking speed	LC-MS (Broad Institute of MIT and Harvard, Cambridge, MA, USA)	plasma 350 (including FAs, AAs, SLs, PCs)	*p* ≤ 0.01 and *q* ≤ 0.3
BLSA (Gonzalez-Freire et al., 2019) *,† [41]	*n* = 504, 49% 70.7 (9.9), all 50+	Not specified	6 m walk	LC-MS/MS (Biocrates, Innsbruck, Austria, p180)	plasma 188 (148 analyzed, including AAs, SLs, PCs, acylcarnitines, biogenic amines, and LPCs)	Spearman rank correlations, *p* < 0.05 and FDR (multiple testing correction); *q* < 0.05
ARIC Study (Li et al., 2018) * [42]	*n* = 383, 52.5% 77.5 (5.5)	White (75%)	4 m walk	triple-quadrupole mass spectrometer (Biocrates, Innsbruck, Austria, p180)	plasma 188 (12 analyzed, including PCs and SLs)	*p* < 0.05
Kyoto University Hospital (Kameda et al., 2020) [43]	*n* = 19 (10 non-frail); 63.2% 84.2 (6.9)	East Asian (Japanese, 100%)	TUG	LC-MS/MS (Thermo Fisher Scientific, Waltham, MA, USA)	whole blood 131 (untargeted, including AAs, acylcarnitines, and lactones)	*p* < 0.05
U.S. Veterans LIFE Study (Lum et al., 2011) * [44]	*n* = 77, 0% 79.2 (4.8), all 70+	Not specified	8 ft walk 400 m walk	MS	plasma 45 (Acylcarnitines only; PCA score)	*p* < 0.05
Singapore Longitudinal Ageing Study Wave 2 (Lu et al., 2020) * [45]	*n* = 189; 63% Sarcopenia:73.9 (5.3), No sarcopenia:72.5 (5.3) Range 65–90	East Asian (Chinese, 100%)	6 m walk	N/A (Bevital Lab, Bergen, Norway)	plasma Not specified (27 analyzed, including AAs)	*p* < 0.05
Geriatric Medicine Department of Beijing Hospital (Meng et al., 2022) * [46]	*n* = 246; 0% Sarcopenia: 80.9 (8.5) Nonsarcopenia: 78.6 (7.4) Range 61–100	East Asian (Chinese, 100%)	6 m walk	LC-MS/MS (Sciex and Agilent, Santa Clara, CA, USA)	Serum Not specified (targeted, including AAs, acylcarnitines, and LPCs)	*p* < 0.05
Mayo Clinic Study of Aging (Wennberg et al., 2018) * [47]	*n* = 340, 38.2% median 80.3, IQR:77.2–83.7 range 70–95	Not specified	GAITRite-5.6 m electronic walk-way	LC/ESI/MS/MS (Sciex, Agilent, Santa Clara, CA, USA)	plasma Not specified (12 analyzed, including SLs)	*p* ≤ 0.05
Bordeaux Centre of the Three-City Study (Frison et al., 2017) * [48]	*n* = 982, 59.1% Low gait speed: 75.5 (4.7) Not low gait:73.6 (4.8) All 65+	Not specified (French, 100%)	6 m walk	GC	plasma Not specified (12 analyzed, FAs only)	*p* < 0.005
Division of Geriatrics of the Department of Internal Medicine of the Asan Medical Center in Seoul, South Korea (Jang et al., 2020) * [49]	*n* = 73, 56.2% robust: 67.6 (6.8) pre-frail: 69.8 (5.9) frail: 70.8 (5.0)	East Asian (South Korean, 100%)	4 m walk	LC-MS/MS	serum 3 (targeted, Kynurenine, tryptophan, and ratio of the two)	*p* < 0.05
National Center of Gerontology (He et al., 2020) * [50]	*n* = 451 (316 non-frail), 47% 75.2 (6.6), all 65+	East Asian (Chinese, 100%)	15 ft walk time for slowness	UPLC-MS/MS (Waters Corp, Milford, MA USA)	plasma 1 (targeted, Trimethylamine N-Oxide only)	*p* < 0.05

Notes: BLSA = Baltimore Longitudinal Study of Aging; ARIC = Atherosclerosis Risk in Communities; MCI = Mild Cognitive Impairment; ESI = Electrospray Ionization; MS = Mass Spectrometry; LC = Liquid Chromatography; GC = Gas Chromatography; UPLC = Ultra Performance LC; LIFE = Learning to Improve Fitness and Function in Elders; ABC = Aging and Body Composition; CHS = Cardiovascular Health Study; MMSE = Mini-Mental State Examination; TUG = Timed Up and Go; PCA = Principal Component Analysis; * = reported cross-sectional associations; † = reported longitudinal associations. Studies are sorted by the number of metabolites examined, from highest to lowest.

## Data Availability

Not applicable.

## Supplementary Materials

The following supporting information can be downloaded at: https://www.mdpi.com/article/10.3390/metabo12040356/s1. Table S1: Associations between metabolites and memory and gait.
